# Chitosan-based nano-sorbents: synthesis, surface modification, characterisation and application in Cd (II), Co (II), Cu (II) and Pb (II) ions removal from wastewater

**DOI:** 10.1038/s41598-023-32847-3

**Published:** 2023-04-13

**Authors:** Dipesh Chandra, Md. Tamzid Hossain Molla, Md. Abul Bashar, Md. Suman Islam, Md. Shameem Ahsan

**Affiliations:** Department of Applied Chemistry and Chemical Engineering, University of Rajshahi, Rajshahi, 6205 Bangladesh

**Keywords:** Environmental sciences, Health care, Nanoscience and technology

## Abstract

In contemplation of treating hazardous industrial wastewater, sodium tripolyphosphate (TPP) and vanillin (V)-modified chitosan-based magnetic nano-sorbents (TPP-CMN and V-CMN) were prepared, and the physical and surface properties of both nano-sorbents were characterised. The results of FE-SEM and XRD showed an average size of between 6.50 and 17.61 nm for Fe_3_O_4_ magnetic nanoparticles. The Physical Property Measurement System (PPMS) was carried out, and the saturation magnetisations for chitosan, Fe_3_O_4_ nanoparticles, TPP-CMN, and V-CMN were 0.153, 67.844, 7.211, and 7.772 emu.g^−^1, respectively. By using multi-point analysis, the BET surface areas of the synthesised TPP-CMN and V-CMN nano-sorbents were found to be 8.75 and 6.96 m^2^/g, respectively. The synthesised TPP-CMN and V-CMN were investigated as effective nano-sorbents to uptake Cd (II), Co (II), Cu (II), and Pb (II) ions, and the results were investigated by AAS. The adsorption process of heavy metals was investigated by the batch equilibrium technique, and the sorption capacity values of Cd (II), Co (II), Cu (II), and Pb (II) ions by TPP-CMN were 91.75, 93.00, 87.25, and 99.96 mg/g. By V-CMN, the values were 92.5, 94.00, 88.75, and 99.89 mg/g, respectively. The equilibrium times for adsorption were found to be 15 minutes for TPP-CMN and 30 minutes for V-CMN nano-sorbents. The adsorption isotherms, kinetics, and thermodynamics were studied to understand the adsorption mechanism. Furthermore, the adsorption of two synthetic dyes and two real wastewater samples was studied and obtained significant results. These nano-sorbents’ simple synthesis, high sorption capability, excellent stability, and recyclability may provide highly efficient and cost-effective nano-sorbents for wastewater treatment.

## Introduction

The environment has recently grown hostile, posing a threat to human health and welfare, as a result of the emission of toxins from industry and sewage from cities. The effluents discharged from industries like tanneries, footwear and leather, electroplating, paint, textiles, when added to urban sewage, may pollute surface water bodies used for drinking and washing, via canals, rivers, and surface run-off^1,2^. Heavy metals, according to Fu et al. have a specific gravity greater than 5 and atomic weights between 63.5 and 200.6^3^. The heavy metals most frequently found in our environment are cadmium (Cd), chromium (Cr), copper (Cu), cobalt (Co), nickel (Ni), zinc (Zn), manganese (Mn), and lead (Pb). These tend to accumulate in the environment, they are unstable and often toxic, so they pose a threat to all living creatures^4^. Heavy metals, hazardous compounds, and dyes abound in industrial discharges. Heavy metals are thought to be the most dangerous because of their potential to accumulate in human bodies and cause significant illnesses and physical problems.

Many methods for removing heavy metals and dyes from water have been proposed recently, including chemical precipitation, ion-exchange, adsorption, membrane filtration, photocatalytic degradation, and electrochemical technologies^3–7^. Adsorption, among these approaches, provides flexibility in design and operation, while producing high-quality treated effluent in many circumstances. Thus, different adsorbents like nanoparticles, nanotubes, activated carbon, etc. have recently been used in experiments for the treatment of wastewater by many researchers^8–13^. Sometimes, adsorbents can also be renewed by an appropriate desorption process^14^. Due to their unique magnetic characteristics, non-toxicity, biocompatibility, and relatively low-cost of manufacture, ferrite nanoparticles have received a lot of attention over the last decade, allowing them to be used in a variety of applications^15^. Magnetic nanoparticles are now vital for the future of nano-medicine, since they can carry and target medications, as well as transport imaging agents to their targets; they can also be used in environmental applications^16–19^. Their surfaces can be functionalised by use of organic compounds such as polymers, giving magnetic nanoparticles new capabilities.

After cellulose, chitin is the world’s most omnipresent nitrogenous natural polysaccharide and is a homopolymer of 1→4 linked 2-acetamido-2-deoxy-β-d-glucopyranose. The biosphere contains vast amounts of chitin, which is found principally in the exoskeleton and internal structure of crustaceans (e.g. prawns, crabs, lobsters, crayfish, krill and barnacles)^20–22^. Chitosan, the alkaline deacetylated product of chitin, is a highly-reactive and more versatile chemical compound than chitin. It is especially effective because it contains amino groups as well as hydroxyl groups in its chemical structure^21^. In an acidic environment, chitosan, as a polyelectrolyte, may form electrostatic complexes^23^. Chitosan is a cationic polysaccharide and its cationic nature in a medium where pH is less than 7 is unique among polysaccharides^24^. The fields with the greatest potential for the application of chitosan are agriculture, water and waste treatment, food and beverages, cosmetics and toiletries, and biopharmaceutics, due to its multifarious properties. Especially important in biopharmaceutics, chitosan is biocompatible, biodegradable, less toxic than other common alternatives, antimicrobial and good at forming films^23^. Alginate/chitosan systems have been used in wound dressings and in bone tissue engineering^25,26^. Especially in agriculture, chitosan can effectively inhibit mycelium growth of pathogens and promote plant resistance to abiotic and biotic stresses. Chitosan thus has a potential to aid in limiting the damage produced by agricultural plant diseases, which damage or destroy many crops every year^27^.

No study has yet been reported in the published literature which tests the ability of chitosan to adsorb a number of heavy metals and synthetic dyes together, using a single adsorbent. Thus, this research explores the potential of chitosan to treat wastewater, containing heavy metals and other organic pollutants, using a small amount of the synthesised nano-sorbents of chitosan. The hypothesis is that such a strategy could be more practical than other alternatives. Here, the fabrication of novel nano-sorbents, based on chitosan-coated magnetic nanoparticles with surface modification via simple processes, was investigated. The as-synthesised TPP-CMN and V-CMN nano-sorbents were characterised and their adsorption properties against Cd (II), Co (II), Cu (II) and Pb (II) ions from aqueous solutions, under varied experimental conditions, were also examined. The reusability and synthetic dye sorption capability of the nano-sorbents was also studied.

## Experimental

### Materials

Anhydrous ferric chloride (FeCl_3_), iron (II) sulfate hexahydrate (FeSO_4_·6H_2_O), cobalt (II) chloride hexahydrate (CoCl_2_·6H_2_O), lead nitrate (Pb(NO_3_)_2_), citric acid monohydrate (C_6_H_8_O_7_·H_2_O) crystal, copper (II) sulfate pentahydrate (CuSO_4_·5H_2_O), acetic acid (CH_3_COOH) glacial 100%, hydrochloric acid (HCl) fuming 37%, sodium hydroxide (NaOH) pellets, and absolute alcohol, were purchased from Merck, a German company. Formaldehyde (CH_3_CHO) solution 37% by weight was purchased from Honeywell Riedel-de Haen Research Chemicals, a German company. Vanillin (C_8_H_8_O_3_) was purchased from BDH Chemicals Ltd., a British company. Cadmium (II) sulfate (3CdSO_4_·8H_2_O), was purchased from Loba Chemie Pvt. Ltd., an Indian company. Sodium tripolyphosphate (Na_5_P_3_O_10_) anhydrous extra pure was purchased from Sisco Research Laboratories Pvt. Ltd., an Indian company. All chemicals used in this research work were of analytical quality and used without further purification. Double-distilled water was used in all the studies for sample synthesis and washing purposes.

### Synthesis of chitosan from waste prawn shell

Waste prawn shells were collected from a fish processing factory in Khulna, Bangladesh and the chitosan was prepared according to the literature^28^, with a slight modification, with an 80.26% degree of deacetylation.

### Hydrothermal synthesis of Fe_3_O_4_ nanoparticles

Magnetite (Fe_3_O_4_) was prepared using the hydrothermal co-precipitation method under alkaline conditions, as Ni et al.^29^ did, with slight modification. Anhydrous FeCl_3_ and FeSO_4_·6H_2_O, with a molar ratio of 1:1, were first dissolved separately in distilled water. The prepared solutions were mixed in an Erlenmeyer flask and stirred for 10 minutes, then sonicated for 10 minutes for good dispersion. Under constant stirring, 10% NaOH, as a precipitating agent, was then added, drop-wise, to the mixed solution, to reach the desired pH value of 12.25. After stirring the solution at 1000 r/min for 1 hour at 50 °C, the homogenous blackish solution was placed into a 200 ml Teflon-lined autoclave, sealed, and then heated at 120 °C for 6 hours. After the reaction, the autoclave was cooled to room temperature, filtered, washed with distilled water and absolute alcohol, and the resulting black precipitates were dried in an oven for 4 hours at 60 °C.

### Synthesis of chitosan coated Fe_3_O_4_ nanoparticles (CMN)

CMN was produced as Ren et al.^30^ did, with slight modification. The magnetite was added to a 3% (w/v) chitosan solution in a ratio of 1:70 (w/v). The dispersion of Fe_3_O_4_ was fully completed by ultrasonic vibration for 20 minutes, followed by the addition of a few drops of formaldehyde. After 4.5 hours, a black gel formed and this was left to dry in the oven at 60 °C for 12 hours. Then the dried product was washed, with 2% acetic acid and then with distilled water, and dried further for 12 hours at 50 °C.

### Synthesis of tripolyphosphate-modified chitosan-coated Fe_3_O_4_ nanoparticles (TPP-CMN)

The prepared CMN, with a ratio of 1:200, was suspended in an aqueous solution of citric acid (6%) for 18 hours under magnetic stirring at 1000 rpm. Then the TPP, as a water-soluble cross-linking agent, was added drop-wise into the now-yellowish opaque solution. After sonication for 15 minutes, the resultant solution was stirred further for 5 hours, and the then whitish solution was filtered, using Whatman No. 1 filter paper and washed several times with distilled water. Finally, the synthesised TPP-CMN was dried at 60 °C for 12 hours and stored in a desiccator, after grinding.

### Synthesis of vanillin-modified chitosan-coated Fe_3_O_4_ nanoparticles (V-CMN)

0.5 g of prepared CMN was suspended, in 100 mL of 0.3 M citric acid solution for 18 hours under magnetic stirring at 1000 rpm. The vanillin, dissolved in alcohol, was then added drop-wise to the now-yellowish opaque solution. After 20 minutes of sonication, the resulting solution was agitated for another 5.5 hours for good dispersion. Then, the solution was filtered through Whatman No. 1 filter paper, rinsed multiple times with distilled water, and dried in an oven at 60 °C for 12 hours. After grinding, the dried V-CMN was stored in a desiccator.

### Chemistry of the synthesis and adsorptive behavior of TPP-CMN and V-CMN nano-sorbents

**Figure 1:**
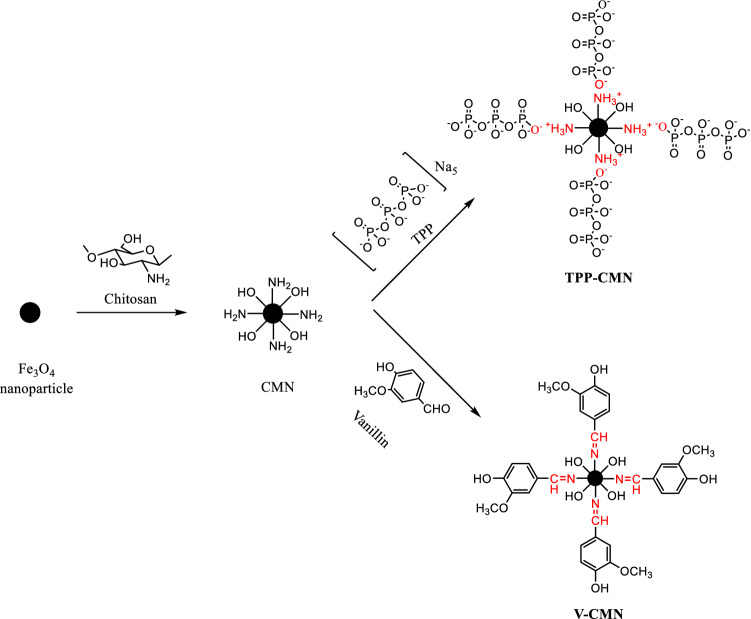
The synthesis route for the production of TPP-CMN and V-CMN nano-sorbents and their adsorption mechanism of heavy metals.

Magnetic nanoparticles require a protective coating to enable them to be used to adsorb heavy metals from wastewater, given their oxidation properties and instability in acidic media^31^. The Fe_3_O_4_ nanoparticles were coated with chitosan polymer to avoid agglomeration, and their surfaces were successively modified by vanillin and sodium tripolyphosphate in two different processes. During the surface modification of CMN, the amine group of the chitosan molecule was successfully protonised by the electrostatic attraction of the anionic moiety of the carboxylic group of the citric acid. When TPP was added to the protonised solution, the electrostatic bonding on the surfaces was permanently fixed. The sonication of the reaction mixture produced excellent dispersion in this step. Besides, the aldehyde group of the vanillin may form a Schiff base ($-\mathrm{N}=\mathrm{CH}-$) with the amine group present on the surface of the CMN. Figure 1 above, is a schematic representation of the overall production of TPP-CMN and V-CMN nano-sorbents. The prepared sorbents exhibited excellent capacity for sorption of Cd (II), Co (II), Cu (II) and Pb (II) ions from the aqueous solutions because of the large number of active sites present on the surface of TPP-CMN and V-CMN. Since the adsorption process was carried out in an environment of an acidic pH, protonation-deprotonation occurred, and then a large number of active sites for adsorption reactions appeared.

### Characterisation of TPP-CMN and V-CMN

The functional groups of the magnetite, chitosan, CMN, TPP-CMN and V-CMN surfaces were analyzed by use of the Fourier Transform Infrared (FTIR) spectrophotometer-3000 Hyperion Microscope Vertex 80. The FTIR spectra were obtained from KBr pressed pellets in the range of 3800–400 cm^−1^ with 1 cm^–1^ resolution. Thermal analysis was performed at temperatures ranging from 30 °C to 800 °C with a heating rate of 10°C/minute using a Perkin Elmer thermal analyzer. The thermogravimetric analysis (TGA) and differential thermogravimetric analysis (DTA) curves for all the samples were obtained in an inert nitrogen atmosphere at a flow rate of 20 mL/minute. The X-ray diffraction (XRD) analysis was accomplished by Smart Lab, Rigaku, Japan. The XRD generator was operated at 40 kV, 40 mA, λ = 1.54Å, and 2θ = 10°‒80°. The diffraction data were recorded with a step width of 0.02° and a time of 0.4 seconds at room temperature. The magnetic measurements of chitosan, Fe_3_O_4_ nanoparticles, TPP-CMN and V-CMN were performed using Physical Property Measurement System (PPMS) at room temperature under the applied magnetic field of 20 kOe. The morphology and size of the products were characterised by using a Schottky Field Emission Scanning Electron Microscope (JSM-7610F, JEOL, Japan). The FE-SEM specimens were coated with platinum using an ion sputtering coating device (JEOL-JEC-3000FC/Auto Fine Coater). The products were then viewed and attached to an EDX unit (for Energy-dispersive X-ray spectroscopy). All samples were examined using an accelerating voltage of 10-15 kV and magnification up to 2,00,000 times. The Brunauer, Emmett, and Teller (BET) method was used to calculate BET-specific surface areas from nitrogen adsorption-desorption isotherms measured by Belsorp mini II (BEL Japan Inc.) at 70 °C in a vacuum for 2 hours. During the adsorption studies, the concentration of metal ions was analyzed using Flame emission atomic absorption spectroscopy (AA-6800, Japan).

### Batch adsorption experiment

A batch adsorption experiment was carried out at 35 °C by agitating the Erlenmeyer flask, containing different metal ion solutions, in a shaker. The effect of initial pH, contact time, metal ion concentration, adsorbent dosage, and temperature were studied to reach the optimum reaction condition. The pH of the initially-prepared metal ion solutions was by the addition of HCl and NaOH. At the end of each experiment, the nano-sorbents were separated magnetically and the post-adsorption solution was collected, filtered using Whatman No.1 filter paper, and the concentration was measured by flame emission atomic absorption spectroscopy (AA-6800, Japan). The metal sorption capacities, ${q}_{e}$ (mg/g) were calculated using the following equation^30^.

1 $${q}_{e}=\frac{{C}_{i}-{C}_{e}}{m}\times V,$$

where, ${C}_{i}$ = initial concentration of the metal ion solution, mg/L; ${C}_{e}$ = equilibrium concentration of the metal ion solution after adsorption, mg/L; $V$ = volume of the solution, L and $m$ = weight of the adsorbent, g.

## Results and discussion

### Characterisation

#### FTIR analysis

**Figure 2:**
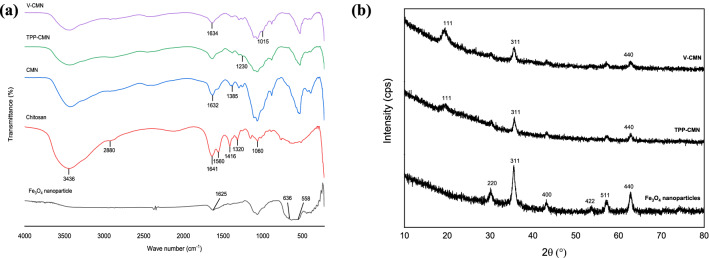
(**a**) FTIR spectra of Fe_3_O_4_ nanoparticles, chitosan, chitosan coated Fe_3_O_4_ nanoparticles, TPP-CMN, V-CMN nano-sorbents, and (**b**) XRD spectra of Fe_3_O_4_ nanoparticles, TPP-CMN, and V-CMN nano-sorbents.

The typical FTIR spectra of the prepared Fe_3_O_4_ nanoparticles, chitosan, CMN, TPP-CMN, and V-CMN are shown in Fig. 2a. The stretching vibration of $\mathrm{Fe}-\mathrm{O}$ bonds in the crystalline lattice of bare ${\mathrm{Fe}}_{3}{\mathrm{O}}_{4}$ is responsible for the absorption peaks at 558 cm^−1^ and 636 cm^−1^. The peak at 1625 cm^−1^ is observed for $\mathrm{O}-\mathrm{H}$ bending vibration^32^. The stretching vibration of $\mathrm{N}-\mathrm{H}$ and $\mathrm{O}-\mathrm{H}$ bonds are responsible for the large peak at 3436 cm^−1^ for chitosan, and the peak at 2880 cm^-1^ represents the stretching vibrations of $\mathrm{C}-\mathrm{H}$ bond. The $\mathrm{N}-\mathrm{H}$ bending vibration in the amide group is found at 1560 cm^–1^ and, the vibration of $\mathrm{O}-\mathrm{H}$ and $\mathrm{C}-\mathrm{H}$ in the ring is responsible for the absorption peaks at 1416 cm^−1^ and 1320 cm^−1^. The peak at 1066 cm^−1^ is the result of the glycosidic linkage ($-\mathrm{C}-\mathrm{O}-\mathrm{C}-$) of the chitosan chain^33,34^. In the case of CMN, a hybrid result of ${\mathrm{Fe}}_{3}{\mathrm{O}}_{4}$ and chitosan is obtained. Here, the peak at 1632 cm^−1^ is weaker than in pure chitosan, confirming the effective coating of chitosan on the ${\mathrm{Fe}}_{3}{\mathrm{O}}_{4}$ surface. The absorption peak at 1385 cm^-1^ demonstrates the CH_3_ symmetrical angular deformation^30^. The considerable shift in the region between 1300 cm^-1^ and 1500 cm^-1^ demonstrates that TPP and vanillin successfully modified the surface of CMN. The peak at 1230 cm^−1^ corresponds to antisymmetric stretching vibrations of $\mathrm{P}=\mathrm{O}$ groups in TPP after the crosslinking process. This finding reveals the production of ionic crosslinking between chitosan-protonated amino groups (${-\mathrm{NH}}_{3}^{+}$) and tri-polyphosphate anionic groups^35^. The peak at 1641 cm^−1^ is ascribed to pure chitosan $\mathrm{C}=\mathrm{O}$ stretching (amide I band) which moved in the V-CMN at 1635 cm^−1^, which signals a stretching vibration of the newly formed $\mathrm{C}=\mathrm{N}$ bond between chitosan and vanillin. The vibration of $\mathrm{O}-{\mathrm{CH}}_{3}$ of vanillin is represented by the peak at 1015 cm^−1^^36,37^.

#### XRD analysis

The XRD patterns of TPP-CMN and V-CMN nano-sorbents, as well as Fe_3_O_4_ nanoparticles, are presented in Fig. 2b. The characteristic diffraction peaks of Fe_3_O_4_ nanoparticles at 30.18°, 35.51°, 43.10°, 53.71°, 57.03°, and 62.74° could be assigned to the [220], [311], [400], [422], [511], and [440] planes of magnetite^30^. In addition, the average crystal size was calculated from the full-width at half-maximum (FWHM) of the magnetite [311] diffraction peak at 2θ = 35⋅51°, using Scherrer’s equation^38^.

2 $$L=\frac{k\lambda }{\beta cos\theta },$$

where, L = average crystallite size, nm; λ = X-ray wavelength, nm; $\beta$= peak width of the diffraction peak profile at half the maximum height resulting from small crystallite size, radians; k = constant related to crystallite shape normally taken as 0.9. The θ can be in degrees or radians.

The crystal sizes were found to be 10.2, 12.9, 12.7, 6.5, 9.0 and 11.5 nm. The lattice parameters (a = b = c =8.38 and α = β = γ = 90°) reveal the inverse cubic spinel structure of magnetite. The XRD pattern of TPP-CMN nano-sorbent shows five characteristic peaks at 20.74°, 35.64°, 57.22°, 62.80°, and 68.6° and the crystal sizes are 1.19, 14.4, 14.0, 10.7, and 1.00 nm. XRD pattern of V-CMN nano-sorbent shows five characteristic peaks at 19.36°, 35.64°, 57.17°, 44.1°, and 62.78° and the crystal sizes are 6.1, 12.0, 9.8, 0.27 and 9.5 nm. The lattice parameters (a = b = c = 8.36 and α = β = γ = 90°) indicate that the modifications do not have any significant effect on the inverse cubic spinel structure of magnetite.

#### Thermal analysis

**Figure 3:**
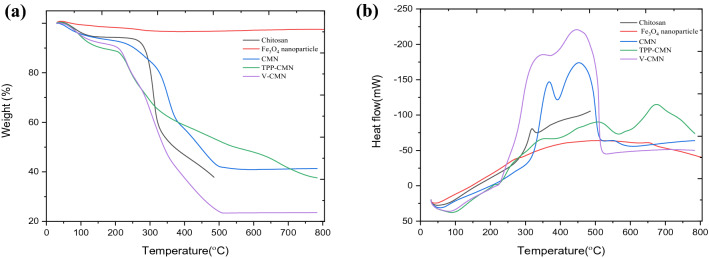
(**a**) TGA curves and (**b**) DTA curves of chitosan, Fe_3_O_4_ nanoparticle, CMN, TPP-CMN and V-CMN.

The thermograms of chitosan, Fe_3_O_4_ nanoparticle, CMN, TPP-CMN and V-CMN obtained by thermogravimetric analysis (TGA) are shown in Fig. 3a below. The first weight loss for chitosan was seen in the temperature range of 35 °C to 100 °C, corresponding to a loss of moisture of about 10%. A non-oxidative thermal degradation occurred at 250 °C, indicating the deacetylation of chitosan and the elimination of volatile products. The TGA curves of CMN show that the weight loss over the temperature range from 30 °C to 800 °C is about 59%. The first decomposition is about 4%, at 100 °C, due to the loss of residual water in the sample. The second decomposition starts at 220 °C and goes to a maximum at 380 °C, with a 35% loss of weight. This might be due to the loss of the chemical water^39^ and the release of chitosan film from the surface of Fe_3_O_4_ mesopores. Finally, at around 500 °C, the polymeric chain of chitosan is broken into char residue containing Fe_3_O_4_ mesopores, resulting in an 18% weight loss. The as-synthesised nano-sorbents (TPP-CMN and V-CMN) contain about 5–6% adsorbed water, which evaporates at a relatively low temperature, around 100 °C. It means that this water is physically-adsorbed and/or weakly hydrogen-bonded to adsorbent molecules. The decomposition accompanying the next 6% weight loss of TPP-CMN starts above 100 °C and it reaches to about 220 °C. Most probably, at this stage, strongly hydrogen-bonded water is released, along with cleavage of the TPP-modified chitosan film from the surface of Fe_3_O_4_. Due to the breakdown of the chitosan-TPP skeleton^40^ and the depolymerisation of chitosan chains by generating char residue, together with Fe_3_O_4_, another weight loss, which begins at 320 °C and reaches a maximum of 50% at 700 °C. Furthermore, at 200 °C the weight loss of V-CMN is about 5%, attributable to the dehydration and the decomposition of vanillin-modified chitosan film from the Fe_3_O_4_ surface. Another weight loss, of 21%, occurred at a temperature range of 320–380 °C, due to the breakdown of Schiff base ($-\mathrm{N}=\mathrm{CH}-$), degradation of pyranose rings through dehydration, as well as deamination, and, ultimately, ring-opening reaction^33,41,42^. Finally, the char residue is attributed to the loss of weight of 25% of the V-CMN adsorbent, at a temperature of 500 °C.

The DTA curve of CMN (Fig. 3b) shows an endothermic peak, at 80 °C, indicating that the chitosan on the surface of Fe_3_O_4_ can be easily hydrated, owing to its high affinity for water; so some bound moisture could not be completely eliminated during drying. Two other exothermic peaks are obtained. The first peak, at 375 °C, corresponds to the depolymerisation of chitosan, by cleavage of the glycoside bond and decomposition of the acetyl and deacetylated groups^43,44^. The DTA curve of TPP-CMN shows a broad endothermic at 98 °C. The peaks at 505 °C and 680 °C reveal the phase transition of Fe_3_O_4_ to γ-Fe_2_O_3_ and α-Fe_2_O_3_, respectively. In the case of V-CMN, an endothermic peak is found at 90 °C and two exothermic peaks are at 350 °C and 450 °C.

3 $$2{\mathrm{Fe}}_{3}{\mathrm{O}}_{4}+\frac{1}{2}{\mathrm{O}}_{2} \to 3 {\upgamma -\mathrm{Fe}}_{2}{\mathrm{O}}_{3},$$

4 $$2{\mathrm{Fe}}_{3}{\mathrm{O}}_{4}+\frac{1}{2}{\mathrm{O}}_{2} \to 3 {\mathrm{\alpha }-\mathrm{Fe}}_{2}{\mathrm{O}}_{3}.$$

#### Magnetic property analysis

**Figure 4:**
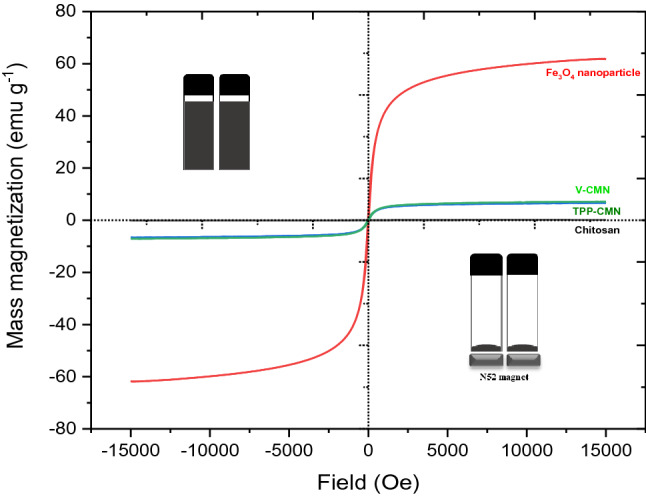
Magnetic hysteresis loop of chitosan, Fe_3_O_4_ nanoparticle, TPP-CMN and V-CMN at room temperature (inset: magnetic separation of TPP-CMN and V-CMN nano-sorbents after treatment of wastewater).

The magnetic properties of Fe_3_O_4_ nanoparticles have a significant impact on their environmental applications. Magnetic experiments are carried out to investigate whether the synthesised TPP-CMN and V-CMN can be used as nano-sorbents. Typical magnetic hysteresis loops are recorded in Fig. 4. There are three parameters, including saturation magnetisation (${M}_{s}$), coercive force (${H}_{c}$), and magnetic remanence (${M}_{r}$), that are detected from the magnetic hysteresis loop, which describes the response capability of magnetic materials to an external magnetic field. The superparamagnetic properties of the synthesised nano-sorbents are implied by the absence of remanence or coercivity at room temperature because superparamagnetic substances lose their magnetisation when the external field is removed, which is supported by the low values of ${H}_{c}$, ${M}_{r}$, and the ${M}_{r}/{M}_{s}$ ratio in the magnetisation curve. The saturation magnetisation (${M}_{s}$) value of the Fe_3_O_4_ nanoparticles is 75-92 emu.g^−1^^45^*.* In this work, the ${M}_{s}$ for chitosan, Fe_3_O_4_ nanoparticles, TPP-CMN, and V-CMN were 0.153, 67.844, 7.211, and 7.772 emu.g^−1^, respectively.

As shown in Fig. 4, chitosan, a well-known natural biopolymer, has no magnetic effect. Although the saturation magnetisation values of TPP-CMN and V-CMN are lower than those of Fe_3_O_4_ nanoparticles, the nano-sorbents could be promptly separated from the water dispersion following treatment by a permanent Neodymium magnet (N52 grade), as shown in the inset in Fig. 4. These lower values of ${M}_{s}$ for TPP-CMN and V-CMN nano-sorbents than for Fe_3_O_4_ nanoparticles imply the existence of coated materials with proper surface modification, in addition to the nanoparticles’ surface disorder. Superparamagnetic particles have dimensions less than 20 nm, as reported in the literature^46^, which confirms the superparamagnetic behavior of Fe_3_O_4_ nanoparticles, TPP-CMN, and V-CMN nano-sorbents.

#### FE-SEM and EDX analysis

**Figure 5:**
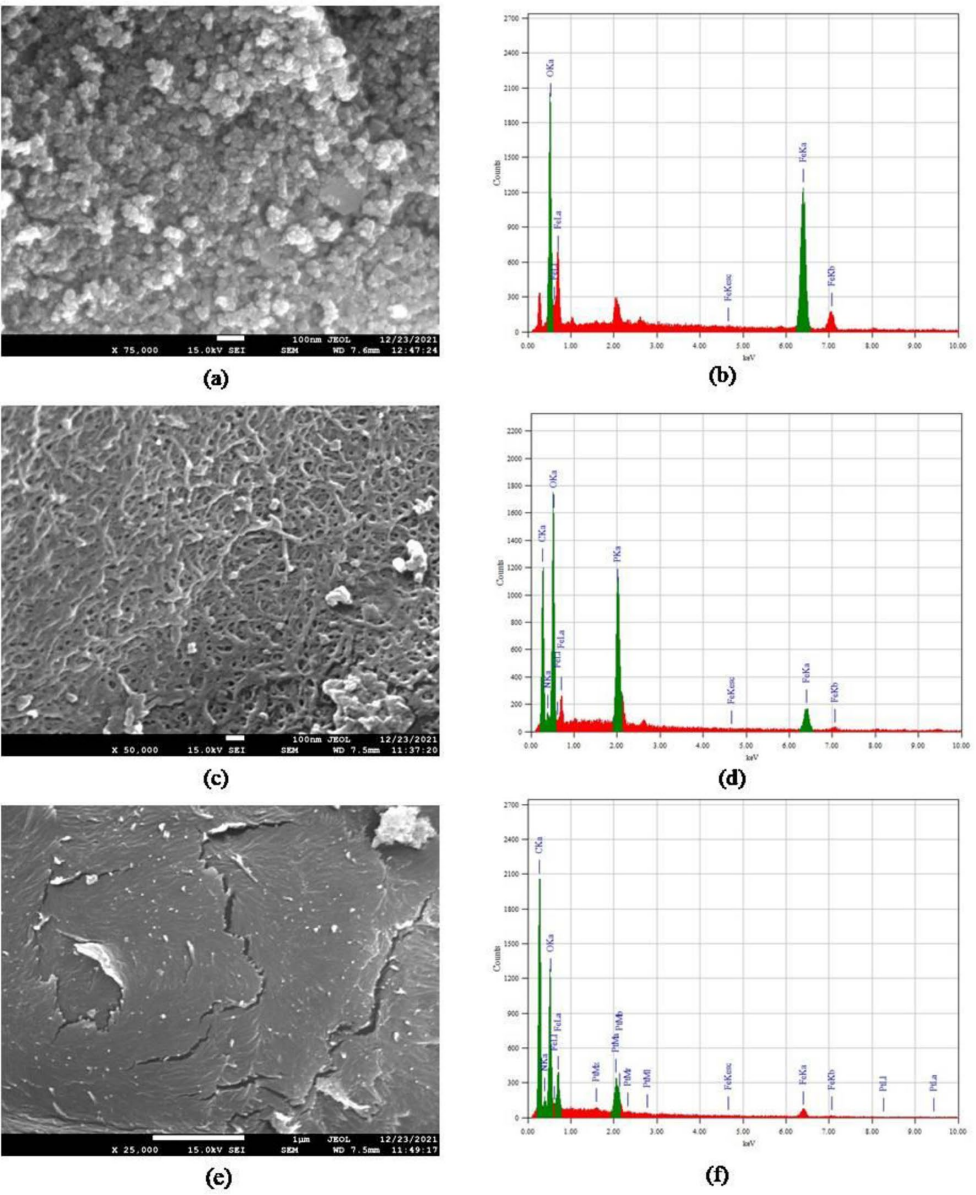
SEM micrographs of (**a**) Fe_3_O_4_ nanoparticles, (**c**) TPP-CMN, (**e**) V-CMN and EDX spectra of (**b**) Fe_3_O_4_ nanoparticles, (**d**) TPP-CMN, (**f**) V-CMN.

The morphological structure of the synthesised products was observed by FE-SEM. Figure 5 presents the micrographs of Fe_3_O_4_ nanoparticles, TPP-CMN, and V-CMN nano-sorbents. The FE-SEM images show different morphological structures and varied particle sizes that confirm the successful coating of chitosan on Fe_3_O_4_ nanoparticles and the surface modification of CMN by TPP and vanillin. The morphograph (Fig. 5a) confirms that magnetite (Fe_3_O_4_) was in a good spherical shape, well-dispersed, and had a diameter ranging from 11.3 to 17.6 nm. The agglomerates are formed from single nanoparticles because individual nanoparticles exhibit magnetic properties. The micrograph of TPP-CMN (Fig. 5c) exhibits a network-like structure with a large number of pores but the layer-type structure of V-CMN (Fig. 5e) does not exhibit any pores on its surface. The variations in morphology lead to changes in physical and chemical properties of the synthesised nano-sorbents, which influence their sorption capacity and the availability of binding sites for the process of heavy metal adsorption.

EDX spectra of Fe_3_O_4_ nanoparticles, TPP-CMN, and V-CMN nano-sorbents, respectively, are shown in Fig. 5b,d,f. The peak of the elements Fe and O, with a small amount of impurities, for Fe_3_O_4_ nanoparticles, is shown in Fig. 5b. The observed percentage of Fe/O value matches well with the amount used in the respective precursors, which indicates that no significant loss occurred during the synthesis. Therefore, it is concluded that this hydrothermal co-precipitation method is very effective, in terms of avoiding loss of elements during synthesis, compared to other conventional methods. The EDX spectrum of TPP-CMN ensured that the nano-sorbents were composed of iron, carbon, nitrogen, oxygen, and phosphorous. And the EDX spectra of V-CMN confirmed that the nano-sorbents were made up of iron, carbon, nitrogen, and oxygen. In short, the modification of the surface of CMN by TPP and vanillin was successful.

#### Surface area of TPP-CMN and V-CMN nano-sorbents

The BET-specific surface area of the TPP-CMN nano-sorbent was found to be 8.75 m^2^/g using multi-point analyses. The total pore volume and average pore diameter were identified as 3.12 × 10^−2^ cm^3^/g and 142.879 Å, respectively. The porosity, based on the skeletal density of 7.8301 g/cm^3^, was calculated as 0.1966 per gram. On the other hand, the BET-specific surface area of the V-CMN nano-sorbent was measured to be 6.96 m^2^/g using multi-point analyses. The total pore volume and average pore diameter were identified as 1.72 × 10^−2^ cm^3^/g and 98.814 Å, respectively. The porosity, based on the skeletal density of 1.6859 g/cm^3^, was calculated as 0.0282 per gram. These two adsorbents, with greater surface area and pore size, provide a large number of sites to which more heavy metals from wastewater can attach.

### Sorption studies

#### Effect of initial solution pH

The initial solution pH is an important operational parameter in metal ion adsorption since it determines the protonation of the functional groups of the adsorbent. pH affects not only protonates but also the speciation of metal ions in solution^30^. When pH rises into the acidic range, the sorption capacity values rise, implying that the appearance of a higher-net-attractive force induces deprotonation at most reaction sites of nano-sorbents. However, a rise in pH values to levels that are too high values might cause M^n+^ to precipitate, which can interrupt the adsorption process. Low metal sorption capacity was found at low pH values, which might be due to competing adsorption between the H_3_O^+^ and M^n+^ ions. Furthermore, the presence of a greater concentration of hydronium ions on adsorbent surfaces appears to create a positive charge, reducing the number of binding sites for metal ions.

**Figure 6:**
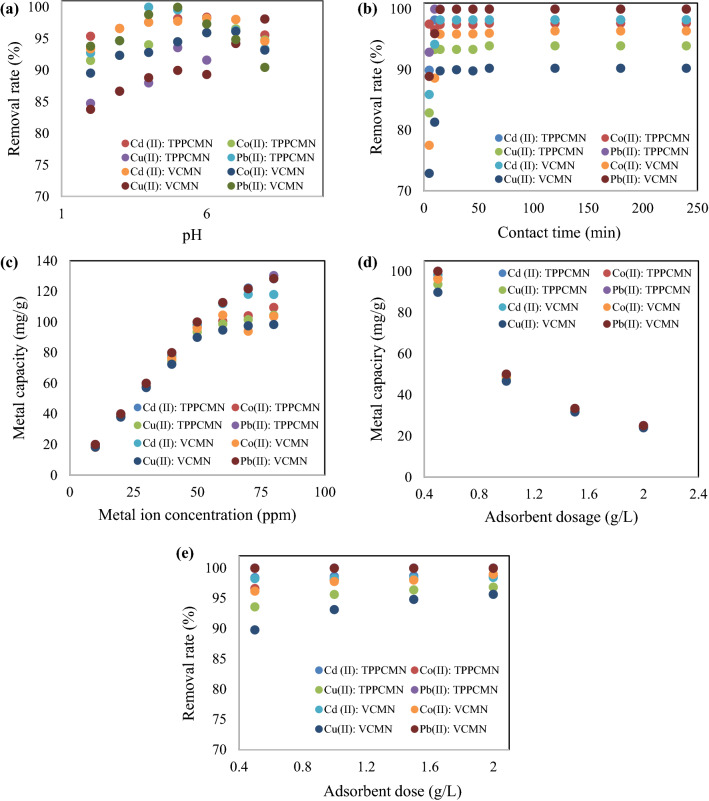
Effect of different operating conditions for the adsorption of Cd (II), Co (II), Cu (II) and Pb (II) ions from aqueous solutions using TPPCMN and VCMN nano-sorbents: (**a**) Initial solution pH, (**b**) Contact time, (**c**) Metal ion concentrations and (**d**,**e**) Adsorbent dosage.

Figure 6a shows that the highest adsorption capacity values of Cd (II) were obtained at pH 6.0 for TPP-CMN and V-CMN nano-sorbents, with an adsorption capacity of 98.31 and 98.00 mg/g, respectively. The highest adsorption capacity values for Co (II) were obtained at pH 7.0 by TPP-CMN and V-CMN nano-sorbents, with adsorption capacity values of 99.25 and 99.00 mg/g, respectively. At pH 5.0, the maximum adsorption capacity was obtained for Cu (II) by TPP-CMN and V-CMN sorbents, with values of 98.00 and 93.26 mg/g. The highest adsorption capacity values of Pb (II) were obtained at pH 4.0 for TPP-CMN and at pH 5.0 for V-CMN nano-sorbents, with an adsorption capacity of 99.98 and 99.96 mg/g, respectively. The nature of the metal ions (including their charge, size and affinity for different sorbents) and adsorbent materials may account for the difference in sorption capacities between metals. Moreover, the excellent adsorption capacity (Pb > Co > Cd > Cu) indicates the potential selectivity of TPP-CMN and V-CMN for Cd (II), Co (II), Cu (II) and Pb (II) ions.

#### Effect of contact time

The effect of contact time was investigated at the optimum pH of the metal ion solutions. Figure 6b shows the findings for Cd (II), Co (II), and Cu (II) metal capacity, in mg/g for TPP-CMN and V-CMN sorbents, at various shaking times. 15 minutes of contact time between metal ions and TPP-CMN and 30 minutes of contact time for V-CMN was enough to achieve equilibrium. This suggests that the adsorption reactions of the examined metal ions by the nano-sorbents could actually follow a two-step mechanism. Surface diffusion would be the initial stage, followed by the sorption process between metal ions and nano-sorbents, which would be the rate-limiting step^47^. The metal sorption capacity steadily increased as the contact time was extended from 5 to 240 minutes. The maximum adsorption of Cd (II), Co (II) and Cu (II) ions was attained at 30 minutes for TPP-CMN and 45 minutes for V-CMN sorbents. After that, the adsorption rate increased slightly, which indicates the potential selectivity of the sorbents. The maximum adsorption values of Cd (II) ions were 87.25 mg/g and 85.25 mg/g for TPP-CMN and V-CMN nano-sorbents, respectively. Co (II) ions exhibited the highest sorption capacity, of 88.25 mg/g and 88.75 mg/g in TPP-CMN and V-CMN nano-sorbents, respectively. The highest adsorption values of Cu (II) ions were 81.25 mg/g and 78.00 mg/g, for TPP-CMN and V-CMN nano-sorbents, respectively. The maximum adsorption values of Pb (II) ions were 99.98 mg/g and 99.92 mg/g, for TPP-CMN and V-CMN nano-sorbents, respectively.

#### Effect of initial metal ion concentration

The sorption capacities of all investigated metal ions were found to be successively enhanced with increases in the concentration from 50 to 100 mg/L (Fig. 6c). The ratio of the available number of metal ions in solution to the number of accessible adsorption sites can also account for the greater metal sorption capacity at higher concentrations. This result is attributable to the presence of a large available number of metal ions compared to a limited number of functional groups on the nano-sorbent surface^48^. The maximum adsorption capacities, using TPP-CMN, were 91.75 mg/g, 93.00 mg/g, 87.26 mg/g and 99.78 mg/g, for Cd (II), Co (II), Cu (II) and Pb (II) ions, respectively. The highest adsorption capacities for V-CMN were 92.94 mg/g, 94.04 mg/g, 88.75 mg/g and 99.93 mg/g, respectively. These findings suggest that the adsorbents exhibit a higher potential for Cd (II), Co (II) and Pb (II) adsorption than Cu (II) adsorption, even at the maximum concentration of 100 mg/L.

#### Effect of adsorbent dosage

The influence of adsorbent dosage was examined by adjusting the quantity of nano-sorbents between 0.5 – 2.0 g/L ± 0.001g/L, under optimum conditions of solution pH and reaction time, for the examined metal ions. It is important to obtain the maximum adsorbent dose in order to optimise the interactions between metal ions and the active sites of the adsorbent^49^. The metal capacity values increased with decreasing the sorbent dosage for all evaluated metal ions (Fig. 6d). This is attributable to the presence of a larger number of metal ions compared to the accessible number of surface-active groups^47^. Although the metal capacity values increased with decreasing the sorbent dose, the removal percentage improved significantly when the doses of nano-sorbents increased from 0.5 to 1.0 g/L and subsequently approached a plateau in the dosage range 1.0–2.0 g/L (Fig. 6e). However, the adsorption capacities of both TPP-CMN and V-CMN nano-sorbents changed only minutely over the dosage, range 0.5–1.0 g/L then decreased markedly with dosage in the range 1.0–2.0 g/L. These results may be explained by the fact that increasing the adsorbent dosage can quadruple the number of accessible adsorption sites, resulting in an increase in removal efficiency. When nearly all the heavy metals in the aqueous solutions had been removed by the adsorbents, however, the number of vacant active adsorption sites increased, but this no longer contributed to the removal percentage. Thus, new sites were there but they were not used, so adsorption capacity decreased. Furthermore, the excessive dose may cause adsorbent aggregation^50^, which may further diminish the adsorption capacities of adsorbents. As a result, at a dosage of 1.0 g/L, TPP-CMN and V-CMN nano-sorbents have a higher removal percentage and a higher adsorption capacity than at 2.0 g/L. For this reason, 1.0 g/L was found to be the optimal dosage for these adsorbents.

#### Effect of ionic strength

**Figure 7:**
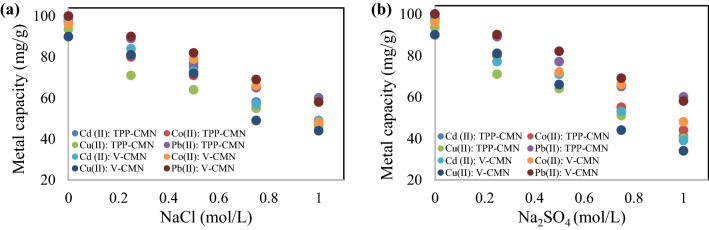
Effect of ionic strength on the adsorption of Cd (II), Co (II), Cu (II), and Pb (II) ions by TPP-CMN and V-CMN nano-sorbents using (**a**) NaCl and (**b**) Na_2_SO_4_.

Ionic strength is an important parameter in assessing the removal of heavy metals by adsorbents via specific and non-specific adsorption. Direct interactions and binding of heavy metals onto the adsorbent occur in specific adsorption, and the removal of Cd (II), Co (II), Cu (II), and Pb (II) ions may not change or increase with increasing ionic strength if the metal ions form inner-sphere surface complexes with functional groups or moieties on the adsorbent surface potentially present. Non-specific heavy metal adsorption, on the other hand, implies that the Cd (II), Co (II), Cu (II), and Pb (II) ions decrease with increasing ionic strength, due to the formation of outer-sphere surface complexes by the metal ions via electrostatic attraction, which influences the adsorption of the retained hydration sphere of the ions to the adsorbent^51^. The specific adsorption of heavy metals is basically not influenced by the solution’s ionic strength, while the non-specific adsorption of metal ions is generally affected by changes in the solution’s ionic strength^52^. The influence of ionic strength parameters on adsorption capacity was investigated at various concentrations of NaCl and Na_2_SO_4_, with the findings presented in Fig. 7a,b.

The adsorption of Cd (II), Co (II), Cu (II), and Pb (II) ions, using TPP-CMN and V-CMN nano-sorbents, was more limited as the competing ion concentrations increased. This could be attributed to Cd(II)/Co(II)/Cu(II)/Pb(II) and Na^+^/Cl^−^/SO_4_^2−^ formation of moieties on the surface of TPP-CMN and V-CMN nano-sorbents through outer-sphere complexes. Furthermore, at higher ionic strengths, competition between Cd(II)/Co(II)/Cu(II)/Pb(II) and Na^+^/Cl^−^/SO_4_^2−^ may reduce the potential energy for migration and heavy metal diffusion to the binding sites of the adsorbed bent, influencing the equilibrium adsorption capacity. These results were consistent with the published literature on this subject^51,53^.

### Kinetic study

**Figure 8:**
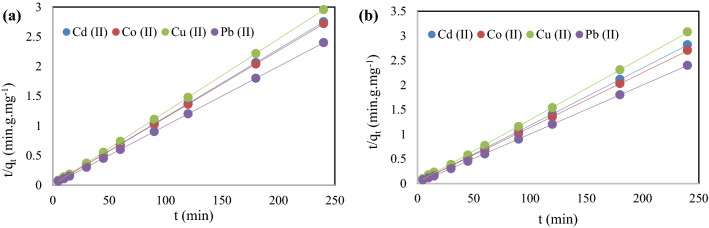
Pseudo-second order kinetic modeling of Cd (II), Co (II), Cu (II), and Pb (II) metal ions using (**a**) TPP-CMN and (**b**) V-CMN nano-sorbents.

To investigate the rate-controlling step in the adsorption mechanism, the kinetic data were analyzed with the help of pseudo-first order and pseudo-second order models. The pseudo-first order model and pseudo-second order model, respectively, are Eq. (5) and Eq. (6)^54^.

5 $$\mathrm{ln}\left({q}_{e}-{q}_{t}\right)=ln{q}_{e}-{K}_{1}t,$$

6 $$\frac{t}{{q}_{t}}=\frac{1}{{K}_{2}{q}_{e}^{2}}+\frac{t}{{q}_{e}},$$

where, ${q}_{t}$ = amount of metal ions adsorbed at time t (mg/g), ${q}_{e}$ = amount of metal ions adsorbed at equilibrium (mg/g), ${K}_{1}$ = rate constant of the pseudo-first order model (min^−1^), ${K}_{2}$ = rate constant of the pseudo-second order model (g.mg^−1^.min^−1^), $t$ = time (min). The adsorption kinetics of Cd (II), Co (II), Cu (II), and Pb (II) metal ions onto TPP-CMN and V-CMN surfaces were studied and listed in Table 1 and the graph produced by the pseudo-second order kinetic model are shown in Fig. 8.

**Table 1:** Kinetic parameters for the adsorption Cd (II), Co (II), Cu (II), and Pb (II) ions.

Sorbents	Metal	q_e,exp_	Pseudo-first order			Pseudo-second order		
			q_e,cal_	K_1_×10^−5^	R^2^	q_e,cal_	K_2_	R^2^
TPP-CMN	Cd (II)	87.25	0.467	− 5.921	0.1332	87.413	0.0267	0.998
	Co (II)	88.25	0.912	− 6.825	0.2206	88.574	0.0157	0.999
	Cu (II)	81.25	0.621	− 5.379	0.1409	81.367	0.0306	0.999
	Pb (II)	99.70	0.693	− 5.133	0.130	100.301	0.02106	0.999
V-CMN	Cd (II)	85.25	1.969	− 7.363	0.2943	85.616	0.0125	0.998
	Co (II)	88.75	2.613	− 9.471	0.3675	89.526	0.6733	0.999
	Cu (II)	78.00	2.329	− 8.396	0.3071	78.616	0.0084	0.997
	Pb (II)	99.91	3.563	− 4.121	0.233	100.505	0.0132	0.999

From the higher statistical linear coefficient values (R^2^) and the consistency of the qe (from experimental) and qe,cal (from pseudo-second order model) values, we can conclude that the pseudo-second order model is more-reliable to predict the kinetic aspects of the adsorption. The adsorption process can be represented in two steps:

1. The transfer of the solute molecules from the aqueous solution to the surface of the nano-sorbents.
2. The diffusion of the adsorbate molecules into the interior pores of the nano-sorbents^55^.

### Isotherm models

The adsorption of studied metals on to various sorbents was investigated using several adsorption isotherm models, including Langmuir’s (Eq. (7)) and Freundlich’s (Eq. (8))^56^.

7 $$\frac{1}{{q}_{e}}=\frac{1}{{q}_{max}}+\frac{1}{{q}_{max}{K}_{L}{C}_{e}},$$

8 $$\mathrm{log}\left({q}_{e}\right)=\mathrm{log}\left({K}_{F}\right)+\frac{1}{n}\mathrm{log}\left({C}_{e}\right),$$

where, ${q}_{e}$= equilibrium concentration of adsorbate (mg/g), ${q}_{max}$= maximum sorption capacity of the sorbent (mg/g), ${C}_{e}$= liquid phase equilibrium concentration of adsorbate (mg/L), ${K}_{L}$= equilibrium constant (L/g), ${K}_{F}$= sorption capacity constant (L/g), *n*= sorption intensity constant.

**Figure 9:**
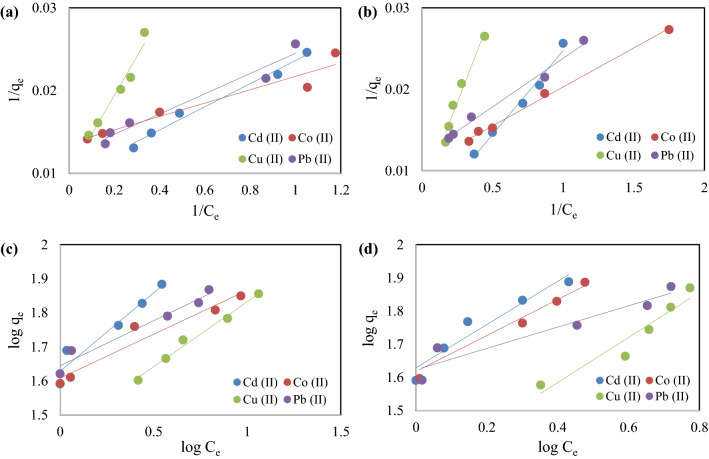
Linear graphs of adsorption isotherms of Cd (II), Co (II), Cu (II), and Pb (II) ions. (**a**) Langmuir’s isotherm model for TPP-CMN, (**b**) Langmuir’s isotherm model for V-CMN, (**c**) Freundlich’s isotherm model for TPP-CMN, and (**d**) Freundlich’s isotherm model for V-CMN.

The linear plots of $1/{q}_{e}$ versus $1/{C}_{e}$ using Langmuir model and $\mathrm{log}\left({q}_{e}\right)$ versus $\mathrm{log}\left({C}_{e}\right)$ using Freundlich’s model are shown in Fig. 9. The entire statistical linear coefficient values (R^2^) are listed in Table 2. The R^2^ values (0<R^2^<1) of the adsorption using TPP-CMN and V-CMN nano-sorbents leads to the conclusion that the adsorption processes are favourable. The identified R^2^ values of the Freundlich model for TPP-CMN were found greater than the values using the Langmuir adsorption isotherm model. But the adsorption onto the V-CMN surface was better predicted by the Langmuir isotherm model.

**Table 2:** Langmuir’s and Freundlich’s isotherm parameters for Cd (II), Co (II), Cu (II), and Pb (II) ions adsorption using TPP-CMN and V-CMN nano-sorbents.

Sorbent	Metal	Langmuir model				Freundlich model		
		q_max_	K_L_	R_L_	R^2^	K_F_	n	R^2^
TPP-CMN	Cd (II)	104.384	0.687	0.018–0.035	0.909	42.906	2.211	0.905
	Co (II)	77.101	1.049	0.012–0.023	0.856	45.254	5.055	0.926
	Cu (II)	141.034	0.115	0.098–0.178	0.732	27.073	2.506	0.941
	Pb (II)	82.782	0.790	0.016–0.031	0.829	42.451	3.788	0.896
V-CMN	Cd (II)	203.666	0.253	0.047–0.090	0.935	42.547	1.536	0.925
	Co (II)	78.864	1.895	0.007–0.013	0.937	48.588	2.818	0.861
	Cu (II)	130.208	0.173	0.067–0.126	0.899	20.553	1.473	0.859
	Pb (II)	81.967	1.045	0.012–0.023	0.942	42.461	3.186	0.886

According to Langmuir’s isotherm model, the equilibrium parameter (a dimensionless constant separation factor) *R*_*L*_ is defined by the following equation-

9 $${R}_{L}=\frac{1}{1+{K}_{L}{C}_{i}},$$

where, C_i_ is the initial concentration of metal ions, mg/L. The R_L_ values (0<R_L_<1) of TPP-CMN and V-CMN, shown in Table 2, suggest that the adsorption processes are favourable. The adsorption features of Cd (II), Co (II), Cu (II) and Pb (II) onto TPP-CMN and V-CMN surfaces might be a case of multilayered and uniform adsorption.

### Thermodynamic study

**Figure 10:**
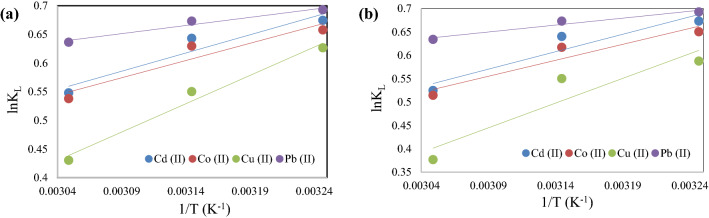
van’t Hoff plots for the adsorption of Cd (II), Co (II), Cu (II) and Pb (II) ions on (**a**) TPP-CMN and (**b**) V-CMN nano-sorbents.

The effect of temperature on the adsorption of Cd (II), Co (II), Cu (II), and Pb (II) ions using TPP-CMN and TPP-V nano-sorbents was also investigated, to calculate its thermodynamic parameters. The adsorption process of the metal ions on to the surface of the nano-sorbents is mainly dependent on the thermodynamic parameters such as the Gibbs free energy change (ΔG°), the enthalpy change (ΔH°) and the entropy change (ΔS°) which were calculated by using the isothermal model (Eq. (10)) and the van’t Hoff model (Eq. (11))^57^:

10 $${\Delta G}^{0}=-RTln{K}_{L},$$

11 $$ln{K}_{L}=\frac{{\Delta S}^{0}}{R}-\frac{{\Delta H}^{0}}{RT},$$

where, R = gas constant (8.314 J mol^−1^ K^−1^), T = temperature (K), K_L_ = equilibrium constant. ΔH° and ΔS° were calculated from the slope and intercept of the van’t Hoff graph of lnK_L_ vs. 1/T (Fig. 10) and reported in Table 3 below.

**Table 3:** Thermodynamic parameters for the adsorption of Cd (II), Co (II), Cu (II), and Pb (II) ions.

Metal ion	T (°C)	TPP-CMN			TPP-V		
		ΔG° (kJ mol^−1^)	ΔH° (kJ mol^−1^)	ΔS° (J mol^−1^ K^−1^)	ΔG° (kJ mol^−1^)	ΔH° (kJ mol^−1^)	ΔS° (J mol^−1^ K^−1^)
Cd (II)	35	− 1.727	− 5.288	− 11.469	− 1.723	− 6.197	− 14.408
	45	− 1.701			− 1.692		
	55	− 1.494			− 1.431		
Co (II)	35	− 1.684	− 5.007	− 10.696	− 1.664	− 5.671	− 12.909
	45	− 1.665			− 1.631		
	55	− 1.467			− 1.402		
Cu (II)	35	− 1.604	− 8.222	− 21.418	− 1.504	− 8.774	− 23.427
	45	− 1.454			− 1.454		
	55	− 1.173			− 1.028		
Pb (II)	35	− 1.774	− 2.364	− 1.891	− 1.773	− 2.457	− 2.191
	45	− 1.780			− 1.779		
	55	− 1.735			− 1.728		

The resultant negative values of ΔG° indicate that all the adsorption processes are spontaneous. Furthermore, the negative values of ΔH° indicate an exothermic reaction and the negative ΔS° values suggest the increasing state of orderliness during the adsorption process at the nano-sorbents/metal-solution interface.

### Recycle performance

The long-term stability and reusability of the adsorbents is an essential aspect in the evaluation of industrial applications since the capacity to recycle inputs contributes significantly to cutting operational costs. The capacity of the synthesised sorbents to adsorb Cd (II), Co (II), Cu (II), and Pb (II) ions from aqueous solutions was evaluated in a continuous operation process for up to 5 cycles under optimised reaction conditions. These optimised reaction conditions were: (volume = 50 ± 1 mL, pH = 5-6, time = 30 ± 1 min, adsorbent dosage = 1.0 ± 0.001 g/L, initial metal ion concentration = 50 ± 1 mg/L). The adsorbents were separated magnetically for the recycle runs, and the equilibrium concentration of the treated solution was determined using atomic absorption spectroscopy following adsorption.

**Figure 11:**
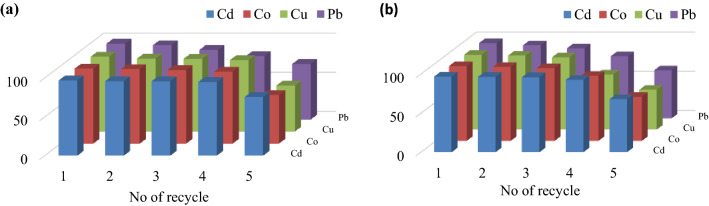
Recycle performance of (**a**) TPP-CMN and (**b**) V-CMN nano-sorbents.

After drying the adsorbent at 60 °C for 1 h, another solution of metal ions was investigated, using the recycled sorbent many times. About 75% of the adsorption capacities of Cd (II), Co (II), Cu (II), and Pb (II) ions were achieved for up to four recycles for both TPP-CMN and V-CMN nano-sorbents (Fig. 11a,b). This might be due to the large number of active sites in the synthesised sorbents for the adsorption of heavy metals, which indicates the great stability of the sorbents. Such stability suggests a significant capacity for recycling, which allows the possibility of industrial applications at a low cost.

### Applicability of TPP-CMN and V-CMN nano-sorbents in removing metal ions from mixed metals in a solution

The applicability of TPP-CMN and V-CMN nano-sorbents in the removal of toxic Cd (II), Co (II), Cu (II), and Pb (II) ions from a solution of mixed metal ions was also investigated, and the obtained results are illustrated by Table 4 below. These results show that the nano-sorbents were highly-effective in removing the toxic substances from the solution, with removal rates all well in excess of 90% for both of the nano-sorbents.

**Table 4:** Applicability of nano-sorbents to remove metal ions from mixed metals in a solution.

Nano-sorbents	Removal rate (%)			
	Cd (II)	Co (II)	Cu (II)	Pb (II)
TPP-CMN	97.06	97.28	95.53	98.32
V-CMN	96.21	96.12	92.91	97.41

### Dye adsorption

Even though the nano-sorbents were made to get rid of heavy metals, they can also get rid of dyes. This is especially true for the chitosan molecule on the surface of Fe_3_O_4_ nanoparticles, which has a strong affinity for dye that has been dissolved in water. In the experiment, the synthesised TPP-CMN and V-CMN nano-sorbents were used for the adsorption of RB 59 and RO 14 dyes from an aqueous solution with a concentration of 30 mg/L. In the case of TPP-CMN, the colours gradually faded away over time, until the solution was almost colourless. The concentrations of the treated solution were measured by UV-Visible spectroscopy. After 6 hours of the experiment, a drastic change occurred. The final concentrations of RB 59 and RO 14 were 1.216 mg/L and 1.126 mg/L, respectively. Almost 96% of the colour of the solutions had been removed. This might be due to the ionic interaction between dye molecules and the adsorbents, as well as the hydrophobic interaction of alkyl groups. But V-CMN did not exhibit better results due to the electrostatic repulsion of the benzene ring present in its structure.

### Treatment of real wastewater

The adsorption efficiency of the synthesised nano-sorbents was investigated with two real wastewater samples from textile-dyeing and footwear factories in Bangladesh, and the results were determined using a photoelectric colourimeter (AE-11M). 250 mL of each wastewater sample, without any pH adjustment, was investigated, with 0.5 g of the synthesised nano-sorbents (TPP-CMN and V-CMN) applied to the 250 mL sub-sample. In both cases, the most significant results were obtained after 1.5 hours of treatment. The adsorption rate of the real wastewater samples was sometimes slower than that in the tests of the model pollutants described above, due to the presence of mixtures of organic pollutants^7^.

### Comparison of the observed data with data from other adsorbents

To confirm the advantage of using TPP-CMN and V-CMN nano-sorbents for the adsorption of Cd (II), Co (II), Cu (II) and Pb (II) ions, the results of heavy metal adsorption using other chitosan-modified adsorbents from published research are shown in Table 5. TPP-CMN and V-CMN exhibit a higher adsorption capacity than every alternative, which might be attributed to the larger specific surface area of the prepared nano-sorbents. Another factor in the better performance of TPP-CMN and V-CMN could be their ease of magnetic separation.

**Table 5:** Comparison of adsorption capacities of various adsorbents for Cd (II), Co (II), Cu (II) and Pb (II) ions from published literature.

Adsorbent name	Metal adsorption capacity (mg/g)				Ref
	Cd (II)	Co (II)	Cu (II)	Pb (II)	
Enhanced chitosan beads supported FeO-nanoparticles	82.6^a^	–	67.2^a^	55.8^a^	^58^
*C. sempervirens*-chitosan microcapsules	65.98^a^	–	67.10^a^	–	^59^
NiO nanoparticles	–	85.56^a^ (149.51)^b^	–	–	^60^
Activated carbon from molasses	–	–	(526.32)^b^	(303.03)^b^	^61^
Physically cross-linked chitosan/sodium alginate/calcium ion double-network hydrogel	81.25^a^ (110.69)^b^	–	70.83^a^ (70.32)^b^	176.50^a^ (278.30)^b^	^62^
**TPP-CMN**	**91.75**^**a**^** (104.38)**^**b**^	**93.00**^**a**^** (77.11)**^**b**^	**87.25**^**a**^** (141.04)**^**b**^	**99.96**^**a**^** (82.78)**	**This study**
**V-CMN**	**92.5**^**a**^** (203.67)**^**b**^	**94.00**^**a**^** (78.87)**^**b**^	**88.75**^**a**^** (130.21)**^**b**^	**99.89**^**a**^** (81.97)**	**This study**

^a^From experimental data.

^b^From Langmuir isotherm model.

The significant values are in bold.

## Conclusions

In the study, TPP-CMN and V-CMN nano-sorbents were synthesised in a simple way and characterised by FTIR, XRD, TGA, PPMS, FE-SEM, EDX and BET analysis techniques. The prepared TPP-CMN and V-CMN nano-sorbents were successfully applied to treat wastewater containing Cd (II), Co (II), Cu (II) and Pb (II) ions both in the laboratory and in real industrial wastewater from footwear and textile-dyeing factories. The results show that the sorbents investigated are better at removing Cd (II), Co (II) and Pb (II) compared with Cu (II) ions, and their metal adsorption capacity is Pb (II) > Co (II) > Cd (II) > Cu (II). Yet removal rates were at or near 90% in every case, far better than any published results found from experiments to remove these metals or ions. This may be attributed to the nature of the metals and the adsorbent materials. A small amount (~1.0 g/L) of the synthesised nano-sorbents was used to obtain these comparatively good results. This might be due to the large number of active sites on the sorbent surface, to which the heavy metal ions are attached by chemical bonding. Recycling the nano-sorbents showed a high adsorption rate (> 80%) was maintained even after recycling the TPP-CMN and V-CMN up to four times. The adsorption equilibrium study for TPP-CMN confirmed the expectations of the Freundlich isotherm model best, while the Langmuir model was found to be a better predictor of V-CMN results. The pseudo-second-order kinetic model was the better predictor of the kinetics of the adsorption processes. The thermodynamic parameters showed an exothermic reaction that became more and more orderly during the adsorption process at the nano-sorbents/metal-solution interface. Finally, the as-prepared nano-sorbents may be effective at removing other metal ions, such as Cr (II), Zn (II), Mn (II), As (II), etc., and also have a high capacity for organic pollutant adsorption.

### Ethics approval

This work didn’t involve research on human subjects or animals covered by ethics regulations.

### Consent to participate

This work didn’t involve human subjects.

## Data Availability

Data is available from the corresponding author on request.
